# Genetic differentiation of the oriental rat flea, *Xenopsylla cheopis*, from two sympatric host species

**DOI:** 10.1186/s13071-018-2903-8

**Published:** 2018-06-08

**Authors:** Fang Zhao, Tongzuo Zhang, Jianping Su, Zuhao Huang, Aiguo Wu, Gonghua Lin

**Affiliations:** 1 School of Life Sciences, Jinggangshan University, Ji’an, 343009 China; 2 Qinghai Provincial Key Laboratory of Animal Ecological Genomics, Xining, 810001 China; 3 Key Laboratory of Adaptation and Evolution of Plateau Biota, Northwest Institute of Plateau Biology, Chinese Academy of Sciences, Xining, 810001 China; 4 Yunnan Institute of Endemic Disease Control and Prevention, Dali, 671000 China

**Keywords:** *Xenopsylla cheopis*, Plague, Microsatellites, Genetic differentiation, Sympatric populations

## Abstract

**Background:**

The oriental rat flea (*Xenopsylla cheopis*), which infests several mammals, primarily rats (*Rattus* spp.), is the most notorious vector of human plague. In this study, we measured the genetic differentiation among populations of fleas from the Asian house rat (*Rattus tanezumi*) and the brown rat (*R. norvegicus*) using microsatellite markers in order to investigate the extent of host-switching in this parasite.

**Results:**

We developed 11 polymorphic microsatellite loci for our study, nine of which showed high potential for inbreeding. AMOVA showed that the majority (84.07%, *P* < 0.001) of the variation was derived from within populations, followed by variation among groups (14.96%, *P* < 0.001); in contrast, variation within groups of populations was nearly absent (0.97%, *P* > 0.05). Analyses of the pairwise fixation index revealed that most of the ten allopatric population pairs but none of the five sympatric population pairs were significantly differentiated. Moreover, based on genetic structure clustering analysis, there was obvious differentiation between allopatric populations but not between sympatric population pairs.

**Conclusions:**

These results indicate the presence of frequent migrations of the oriental rat flea between the sympatric Asian house rat and brown rat, causing a high rate of gene flow and limited genetic differentiation. We suggest that there is no clear boundary limiting the migration of oriental rat fleas between the two hosts, and thus both rat species should be monitored equally for the purposes of plague prevention and control.

## Background

The bubonic plague, caused by the bacterium *Yersinia pestis,* is the underlying cause of several of the worst pandemics in human history. There have been three major historical plague pandemics that caused a total of 200 million deaths: the first occurred in the area around the Mediterranean Sea in the 6th century, the second occurred in Europe in the 14th century, and the third started in China during the middle of the 19th century and from there spread worldwide [1]. Even at present, plague remains a threat, with outbreaks still occurring in many parts of the world, especially Africa [2]. Plague transmission from rodents to humans is primarily due to the bite of flea vectors [3, 4].

China has one of the highest rates of plague incidences. There are 12 natural plague epidemic foci, involving 19 provinces and covering an area of 143.45 km^2^ [5]; the Yunnan Province is the most active amongst these foci. For example, Yunnan accounted for approximately 60% of the total human-related plague cases in China from 1986 to 2005 [6]. In Yunnan, eight species of fleas [including *Xenopsylla cheopis* (the oriental rat flea), *Leptopsylla segnis* and *Monopsyllus anisus*] have been recorded as infected by *Y. pestis*, the oriental rat fleas contributing over 90% of the total number of infected flea samples [7]. The main hosts of oriental rat fleas in the province were Asian house rat (*Rattus tanezumi*) and brown rat (*R. norvegicus*) [8] and the fleas as well as plague were distributed almost all over the province [5]. Every year, regular plague surveillance is conducted by the local government. At each monitoring site, rodents are randomly trapped irrespective of their taxonomic status, but the statistical indices such as rodent density (animals caught / number of traps set during a specific time period) and flea index (number of fleas / number of hosts) are calculated based on each rodent species [9].

Many plague outbreaks have involved fleas that are able to infect multiple host species. Although it is widely accepted that flea switching among different sympatric hosts increases plague prevalence [7], few studies have quantified the extent of switching among hosts. The oriental rat flea parasitizes many small mammalian species (mainly rats) and is the most notorious of the plague vectors [10, 11]. Similarly, two of the most important reservoir hosts for human plague in Asian countries are the Asian house rat and brown rat [7]. In many sub-tropical areas, these two rat species are sympatrically distributed, providing a suitable model for analyzing the oriental rat flea host-switching.

Microsatellites are highly polymorphic molecular markers that are frequently used in population genetic analyses to assess levels of relatedness among populations [12]. In this study, we trapped Asian house rats and brown rats from Yunnan and collected oriental rat fleas from the two sympatric rat species. We then used microsatellite markers to measure the genetic differentiation between oriental rat flea populations from the two hosts to determine the extent of host-switching in this flea. The aim of this study is to show whether there is significant genetic differentiation between flea populations and if so, what the potential factors are that influence genetic differentiation of the fleas.

## Methods

### Sampling

**Fig. 1:**
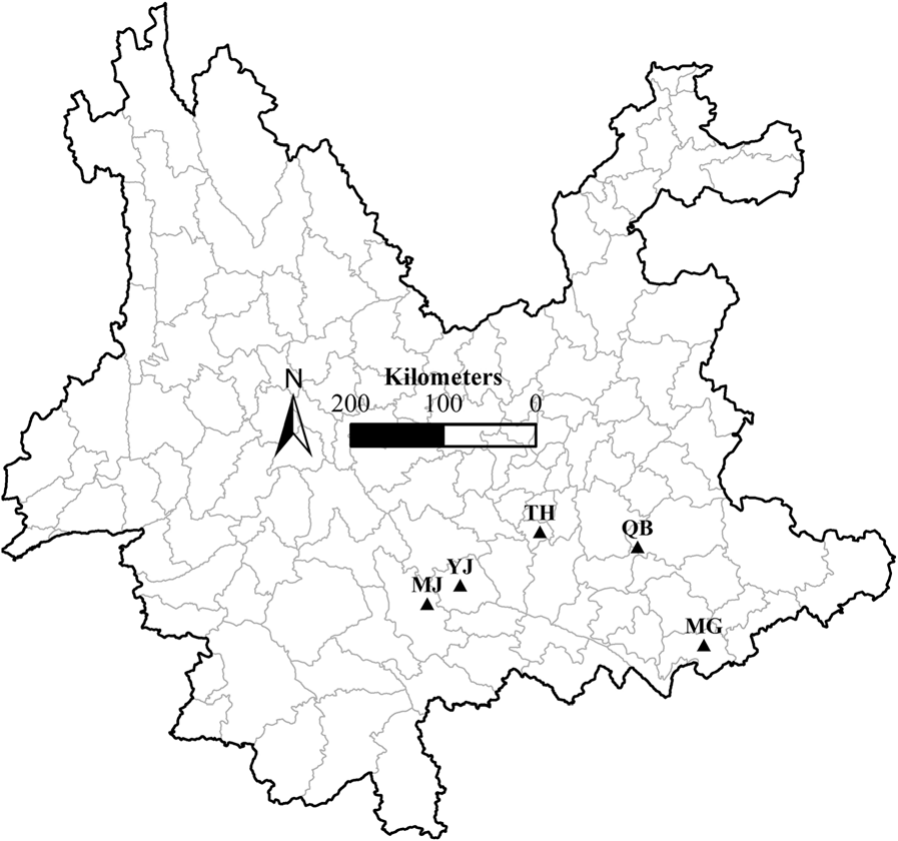
Sampling sites distribution in Yunnan, China

We trapped live rats using trap cages in the houses of local residents from five sites in Yunnan province, China (Table 1, Fig. 1). The geographical information for each sampling site was recorded using an Etrex Global Positioning System (GPS) unit (Garmin, Taipei, China). Each trapped rat was classified according to species by its morphological features and immediately placed into a plastic bag filled with chloroform for 10 min. Dead fleas were collected and stored in 75% alcohol. Muscle samples from each rat were also collected and fixed in 95% alcohol. All captured small mammals were burned after sacrificing and collecting fleas and muscle samples. The taxonomic position of each flea was confirmed under light microscopy in the laboratory. One oriental rat flea was randomly selected from each rat for population genetic analysis. Finally, considering five geographical sites and two hosts, a total of ten flea populations were collected, including 102 fleas respectively collected from 102 rat individuals. We defined two types of relationships between these populations: allopatric (from the same host species at different geographical sites) and sympatric (from different host species at the same geographical site).

**Table 1:** Geographical information and sample size of rat fleas

County	Longitude	Latitude	*R. tanezumi* (*n*)	*R. norvegicus* (*n*)
Maguan (MG)	104°20'29"	23°00'43"	14	15
Mojiang (MJ)	101°39'13"	23°24'55"	13	13
Qiubei (QB)	103°41'54"	23°58'11"	7	10
Tonghai (TH)	102°44'58"	24°07'04"	6	15
Yuanjiang (YJ)	101°58'32"	23°35'38"	3	6

*Note*: the 102 fleas were collected from 102 rat individuals

### Microsatellite primer development

Microsatellites were developed using a traditional isolation protocol [13]. Briefly, the total genomic DNA of 10 fleas (collected from a single rat) was extracted using a DNeasy tissue kit (Qiagen, Hilden, Germany) and was digested with *EcoR*I and *Hind*III. Adaptors containing *EcoR*I and *Mse*I sites were ligated onto the fragments using T4 ligase (Roche, Basel, Switzerland). Enrichment for the motifs (AG)15 and (GT)15 was performed using Streptavidin MagneSphere Paramagnetic Particles (Promega, Madison, WI, USA). Enriched DNA was PCR-amplified and cloned using the pMD18-T Vector System Kit (TaKaRa, Tokyo, Japan). Following β-galactosidase screening, ~100 plasmids were isolated, and cloned inserts were amplified and sequenced using M13 universal primers on an automated ABI 3730xl DNA Analyzer (Applied Biosystems Inc., Carlsbad, CA, USA). Primer sequences were designed by Primers 5 (PREMIER Biosoft, Palo Alto, CA, USA) for 40 loci and were tested for their ability to produce a readable pattern using genomic DNA samples from 10 randomly selected fleas. Finally, 11 primer pairs were selected as working primers. The primer sequences and repeat motif information for all 11 microsatellite loci are listed in Table 2.

**Table 2:** Primer sequence, repeat motif, no. of alleles, inbreeding coefficient, Hardy-Weinberg test, and neutrality test of each locus

Locus	Primer sequence (5'→3')		Repeat motif	No. of alleles	*F*_*IS*_	Hardy-Weinberg test			Neutrality test		
	Forward	Reverse				*H*_*o*_	*H*_*e*_	*P*	*F*_*o*_	L95	U95
P5	TATACCAAATTTCACACAGTT	ATATTCTTAGGAATCCCAGTT	(CT)12	4	0.1251	0.4216	0.6197	< 0.0001	0.3834	0.3212	0.9424
P6	TTAATAGGCATATAATTCAAA	GAATTCCCATCACAACCCATA	(CT)10	7	0.3581	0.1863	0.3282	< 0.0001	0.6734	0.2241	0.7653
P12	TGACTTCCGTGTATCACTAAC	CTAAGGACAAATAAATATGCC	(AG)9	6	01829	0.3922	0.6403	< 0.0001	0.3628	0.2389	0.8439
P15	CCATTTTTCGTACCTGCTACC	GTCAAGAAGAGGACTCGCTGA	(TC)5(TC)4	5	0.6273	0.1275	0.4329	< 0.0001	0.5693	0.2786	0.9051
P19	TTGGTGAGATATAAGTGTGTG	CAAGAAATTGATAACATTTTC	(TC)20	12	0.2313	0.5000	0.8154	< 0.0001	0.1886	0.1419	0.5329
P21	GATCAGGTGTTTCGAGTTATC	TTATTATATATGCTCTTGCGC	(CT)13...(TC)6	5	0.3796	0.2647	0.4430	< 0.0001	0.5592	0.2656	0.8964
P24	AAAAATTGTCGAAAGAAGACA	TTAGAGAAATCGTTGAGGAAA	(GT)7	4	-0.1344	0.3824	0.5014	0.0007	0.5011	0.3287	0.9518
P27	TCATACTAAAATTGAAGAAGG	ACGTAAATACGAATCGAACAC	(TG)6	7	-0.2196	0.5588	0.5143	< 0.0001	0.4882	0.2159	0.8001
P29	TGAATTAGAGTTGCCAAACGG	AATTCGCATAACAATCGCCTA	(GT)3...(GT)6	2	0.0855	0.1863	0.2011	0.6090	0.7999	0.5048	0.9902
P33	ATAAGGAAGTAACCAAAAGGG	TCATCAACAATCAAAACGAAG	(TG)6	2	0.0636	0.3137	0.4090	0.0271	0.5930	0.5024	0.9902
P36	GTGAGGTTCAAAGTCGGGTAT	AATAAAAATCGGTTCAGCAGT	(TG)8	7	0.1668	0.4706	0.6047	0.0006	0.3983	0.2155	0.7537

*Abbreviations*: *F*_*IS*_ inbreeding coefficient, *H*_*o*_ observed heterozygosity, *H*_*e*_ expected heterozygosity, *F*_*o*_ observed frequency, *L95* 95% lower boundary of *F*_*o*_, *U95*, 95% upper boundary of *F*_*o*_

### Microsatellite typing

Total DNA from each flea was extracted using the DNeasy tissue kit. The 5' end of each forward primer was tagged with a FAM fluorophore. All 20 μl PCR reactions were performed using an ABI Veriti96 PCR kit (Applied Biosystems Inc., Massachusetts, USA) and contained 14.8 μl of ddH_2_O, 0.4 μl of dNTPs, 2 μl of buffer, 0.3 μl of each F/R primer, 2 μl of template DNA, and 0.2 μl of Taq DNA polymerase. Reaction mixtures were denatured at 94 °C for 5 min and subjected to 35 cycles of 94 °C for 30 s, 54 °C for 35 s, and 72 °C for 40 s, followed by a final extension step of 72 °C for 3 min. PCR products were separated using a non-denaturing polyacrylamide (8%) gel. GeneScan was used for polymorphism detection on an ABI 3730xl DNA Analyzer system (Sangon, Shanghai, China). Null alleles, scoring errors, and large allelic drop-outs in the microsatellite data were checked using GeneMarker v.1.9.1 [14].

### Data analysis

The observed heterozygosity (*H*_*o*_) and expected heterozygosity (*H*_*e*_) of each microsatellite locus were determined for all samples, and tests of Hardy-Weinberg equilibrium (HWE) were carried out using Arlequin v.3.11 [15]. Tests to measure departures from neutrality were performed and the inbreeding coefficients (*F*_*IS*_) of the 11 loci were analyzed using POPGene v.1.32 [16]. AMOVA (analysis of molecular variance) and genetic differentiation (*F*_*ST*_) calculations were also conducted in Arlequin. For AMOVA, we defined three levels of genetic structure: among groups, namely among the five allopatric sites; within groups of populations, namely between sympatric populations of the five allopatric sites; and within populations, namely within each of the ten populations. For *F*_*ST*_ analysis, we only calculated ten allopatric and five sympatric population pairs. We also calculated the geographical distances between each pair of allopatric sampling sites using ArcGIS v.10.5 (ESRI Inc., Redlands, CA, USA) and tested for correlations between geographical distances and allopatric *F*_*ST*_ values using SPSS v.25.0 (IBM Corp., New York, NY, USA).

The software program STRUCTURE v.2.3.4 [17] was used to estimate the number of populations (*K*) with 20,000 iterations used for burn-in. Probability estimates were determined using 100,000 Markov chain Monte Carlo (MCMC) iterations. Values of *K* were set from 1 to 10 with five runs for each value under an admixture model. In addition, the software programs Structure harvester [18], CLUMPP v.1.1.2 [19], and Distruct v.1.1 [20] were used for repeat sampling and to create a graphical display of the oriental rat flea population structure.

## Results

### General variation information

All 11 of the newly developed microsatellite loci exhibited polymorphisms among the flea samples. A total of 61 alleles were detected at the 11 loci, among which the p19 locus exhibited the highest allelic richness with 12 alleles. All loci except for p29 significantly deviated from Hardy-Weinberg equilibrium (*P* < 0.01) (Table 2). At all loci except for p27, the *H*_*o*_ was lower than the corresponding *H*_*e*_. The *F*_*IS*_ values of nine loci (all except for p27 and p24) were positive. The observed frequency (*F*_*o*_) of each locus was distributed within the range of the 95% confidence interval of the mean.

### Genetic differentiation

AMOVA revealed that the majority (84.07%) of variation was derived from within populations, namely within the ten flea populations (*P* < 0.001). Variation among groups, namely among the five geographical groups, accounted for 14.96% of the total variation (*P* < 0.001). However, variation within groups of populations, namely within five geographical groups of two sympatric hosts, accounted for only 0.97% of the total variation and was not a significant source of variation (*P* > 0.05) (Table 3).

**Table 3:** Results of AMOVA analysis (among groups, among the five allopatric sites; among populations within groups, between sympatric populations within the five allopatric sites; within populations, within each of the ten populations)

Source of variation	*df*	Sum of squares	Variance components	Percentage of variation (%)	*P*
Among groups	4	79.468	0.42668	14.96	0.00098
Among populations within groups	5	14.659	0.02766	0.97	0.45455
Within populations	194	465.118	2.39752	84.07	< 0.00001

Analysis of the pairwise fixation indexes of the 11 loci revealed that, for the fleas from the Asian house rat, seven out of 10 allopatric population pairs were significantly differentiated, while for the fleas from the brown rat, eight out of 10 allopatric population pairs were significantly differentiated (Table 4). Interestingly, none of the five sympatric population pairs were significantly differentiated (Table 5). Pearson correlations showed that the allopatric *F*_*ST*_ values of fleas from Asian house rats (*R* = 0.808, *P* = 0.005) and brown rats (*R* = 0.654, *P* = 0.040) were significantly correlated with the geographical distance between populations.

**Table 4:** Sample size, geographical distance, fixation index (*F*_*ST*_) and *P*-values among populations

Pairwise comparison	Distance (km)	*R. tanezumi* as host			*R. norvegicus* as host		
		*n*	*F*_*ST*_	*P*	*n*	*F*_*ST*_	*P*
MG *vs* MJ	278.72	27	0.3180	< 0.0001	28	0.2243	< 0.0001
MG *vs* QB	124.77	21	0.1491	< 0.0001	25	0.1727	< 0.0001
MG *vs* TH	203.50	20	0.2040	< 0.0001	30	0.1567	< 0.0001
MG *vs* YJ	250.44	17	0.4204	0.0023	21	0.3049	< 0.0001
MJ *vs* QB	217.40	20	0.1150	0.0068	23	0.0434	0.0713
MJ *vs* TH	136.13	19	0.0665	0.0566	28	0.0526	0.0039
MJ *vs* YJ	38.37	16	0.0445	0.2630	19	0.0711	0.0491
QB *vs* TH	97.90	13	0.0461	0.2532	25	0.0196	0.1623
QB *vs* YJ	180.45	10	0.2166	0.0155	16	0.1468	0.0013
TH *vs* YJ	97.88	9	0.1388	0.0485	21	0.0926	0.0019

**Table 5:** Fixation index and *P*-values between sympatric flea populations

Site	*n*	*F*_*ST*_	*P*
MG	29	0.0335	0.0636
MJ	26	0.0104	0.4622
QB	17	-0.0257	0.9735
TH	21	-0.0117	0.8042
YJ	9	0.0947	0.1402

**Fig. 2:**
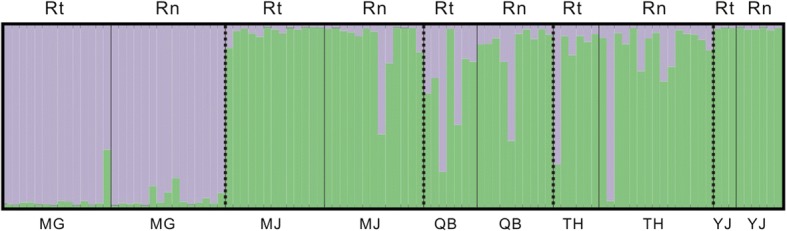
Histogram of the STRUCTURE analysis for the model with *K* = 2. Each color corresponds to a suggest cluster, and a vertical bar represents a single individual. Labels above the histogram correspond to two host species (Rt, *R. tanezumi*; Rn, *R. norvegicus*) and that below the histogram represent five geographical sites

STRUCTURE analysis showed that in the optimal model, *K* = 2. Clustering results showed that there were considerable structural differences among the five allopatric populations. Specifically, the MG population pairs were more distinct than the other four population pairs (Fig. 2), especially compared to the YJ and MJ pairs, probably due to the more distant of MG from the other locations (214.36 km) than those for any of other four locations (≤ 167.66 km) (Table 4). In contrast, few differences were found from each pair of sympatric populations (Fig. 2). These results are consistent with the findings of the earlier fixation index analysis (Table 4).

## Discussion

In the current study, we used 11 newly developed microsatellite loci to assess genetic variation among flea populations. First, neutrality tests showed that the *F*_*o*_ of each locus was distributed within the range of the 95% confidence interval of the mean, indicating that our markers are suitable for population genetic studies. The results of AMOVA as well as *F*_*ST*_, structure, and cluster analysis showed that there was significant genetic differentiation among the five allopatric flea populations, indicating that the markers we used were powerful for analyzing the allopatric population genetics of fleas. Moreover, we detected a significant isolation-by-distance pattern among allopatric oriental rat flea populations, in each of the two host species individually. Such a pattern was not supported by a previous study of the prairie dog flea (*Oropsylla hirsuta*) [21], in which the authors argued that some hosts other than prairie dogs (*Cynomys ludovicianus*) may play an important role in dispersing *O. hirsuta*. According to this hypothesis, the oriental rat flea could be considered to have considerable a close relationship with its *Rattus* hosts. One may note that, since the oriental rat flea could also infest more than one host species, one rat might also influence the spread of fleas from the other rat species (and *vice versa*). We suggest that it will not happen in our oriental rat flea case, because the two house rats have rather similar biological and ecological habits [22].

It should be emphasized that, although there was significant differentiation among the allopatric flea populations, none of the five sympatric population pairs were significantly differentiated. The oriental rat flea parasitizes many genera of rodents inside/outside houses but prefers *Rattus* spp. [23], of which the most widely distributed in Yunnan are the Asian house rat and brown rat [24, 25]. The distributions of these two rat species frequently overlap owing to their similar lifestyle habits [26, 27]. Thus, we speculate that the oriental rat flea ectoparasite possesses plenty of opportunities to transfer to new hosts that may appear within a range of hundreds of miles. In addition, the oriental rat flea can survive up to six weeks without a host, thus providing enough time to allow it to migrate from an old host to a new host [28]. Moreover, the fact that most of these loci exhibited positive inbreeding coefficients and lower observed than expected heterozygosity (*H*_*o*_ < *H*_*e*_) supports to some degree the possibility of inbreeding among the fleas [29]. As a result, it is possible that sympatric migrations have synchronously caused gene flow between fleas from different host species. Based on our results, it is reasonable to conclude that there is frequent migration of the oriental rat flea between the sympatric Asian house rat and brown rat, leading to a high rate of gene flow and limited genetic differentiation.

As mentioned above, the oriental rat flea is the predominant vector of bubonic plague. Although the study area, Yunnan, is the likely site for the origin of the third plague pandemic [1], the epidemiology of the oriental rat flea in this region has not been well studied. Our results provide important fundamental information for the prevention and control of plague in similar areas. For example, for a long time, many experts believed the Asian house rat to be the main host contributing to the high plague prevalence in Yunnan. However, others now believe that the brown rat plays a key role in the prevalence of plague in many regions in which it is distributed [30]. Based on the results of our study, we suggest that there is no clear boundary limiting migration between these two hosts, as evidenced by the high rate of migration of the oriental rat flea. Hence, both rat species should be monitored equally for the purposes of plague prevention and control.

## Conclusions

While it is widely accepted that flea switching among different sympatric hosts increases plague prevalence, little is known about the extent of switching among hosts. In this study, we measured the genetic differentiation among populations of fleas from two sympatric rat hosts using microsatellite markers. The AMOVA results showed that the majority of the variation was derived from within populations while variation between sympatric populations within the five allopatric sites was nearly absent. Analyses of the pairwise fixation index revealed that most of the ten allopatric population pairs but none of the five sympatric population pairs were significantly differentiated. Moreover, based on genetic structure clustering analysis, there was obvious differentiation between allopatric populations but not between sympatric population pairs. These results indicate the presence of frequent migrations of the oriental rat flea between the sympatric Asian house rat and brown rat, causing a high rate of gene flow and limited genetic differentiation. We suggest that there is no clear boundary limiting the migration of oriental rat fleas between the two hosts, and thus both rat species should be monitored equally for the purposes of plague prevention and control.

## Abbreviations

*F*_*IS*_: inbreeding coefficient
*F*_*o*_: observed frequency
*H*_*e*_: expected heterozygosity
*H*_*o*_: observed heterozygosity
L95: 95% lower boundary of *F*_*o*_
Rn: *Rattus norvegicus*
Rt: *Rattus tanezumi*
U95: 95% upper boundary of *F*_*o*_
